# Mechanotransduction, cellular biophotonic activity, and signaling patterns for tissue regeneration

**DOI:** 10.1016/j.jbc.2024.107847

**Published:** 2024-09-30

**Authors:** Claudia Cavallini, Elena Olivi, Riccardo Tassinari, Carlo Ventura

**Affiliations:** 1 ELDOR Lab, Bologna, Italy; 2 Department of Medical and Surgical Sciences (DIMEC), University of Bologna, Bologna, Italy

**Keywords:** cell signaling, development, photobiology, stem cells, regenerative medicine

## Abstract

Signaling molecules exhibit mechanical oscillations, entailing precise vibrational directionalities. These steering signatures have profound functional implications and are intimately connected with the onset of molecular electric oscillations and biophoton emission. We discuss biophotonic activity as a form of endogenous photobiomodulation, orchestrating the mechano-sensing/-transduction in signaling players. We focus on exogenous photobiomodulation in the form of pulsed wave modulation of selected light wavelengths to direct endogenous biophotonic activity and molecular cellular dynamics. We highlight the relevance of this strategy to target and reprogram the developmental potential of tissue-resident stem cells in damaged tissues, affording precision regenerative medicine without the need for cell or tissue transplantation.

Vibration in signaling molecules is not confined within the boundaries of nanomechanics (1, 2), which refers to the study of the mechanical properties and behaviors of materials at the nanoscale, where quantum effects and surface phenomena significantly influence material behavior. The presence of electrically charged amino acids turns proteins into electromechanical actuators, with high-frequency electromagnetic and mechanical oscillations sharing a common megahertz (MHz) frequency domain (3). For some proteins, electromagnetic and mechanical oscillations may have a common time or frequency region, where both electromagnetic and mechanical oscillations merge, with the crucial implication that we can manipulate one another (3).

In biology, nanomechanics focuses on understanding how cells, proteins, DNA, and other biomolecules respond to mechanical forces, which is crucial for processes like cell division, motility, and signaling. The experimental data presented lead us to hypothesize that protein nanomechanics may provide the sensing interface to transduce driven electromagnetic and mechanical signals into precise electromechanical profiles, and molecular developmental pathways.

Mechanotransduction refers to the process by which cells convert mechanical stimuli into biochemical signals. This involves the sensing of mechanical forces by cellular structures, such as the cell membrane, cytoskeleton, and extracellular matrix (ECM), and their translation into molecular events that regulate various cellular functions. Mechanotransduction is critical for processes such as cell growth, differentiation, migration, and response to the physical environment. We will discuss cellular mechanotransduction as an emerging signaling property resulting from intrinsic molecular vibration, entailing mechanical, and electromagnetic (including light) patterns with molecular signaling and developmental capabilities. We will focus on the functional implications of intramolecular vibrational directionality (as discussed below), and the resulting biophoton emission, highlighting the chance of using light to finely tune the nanomechanical properties and mechanotransduction potential in signaling players of cell growth and differentiation. We will finally discuss using interchangeable nanomechanical and light-emitting/sensing profiles of cellular proteins to foster a deeper understanding of growth regulatory mechanisms and develop novel approaches in regenerative and precision medicine.

## Intramolecular vibrational directionality: Electric and biophoton dynamics in proteins

Long-range collective vibrations occur in proteins (4). The use of anisotropic terahertz microscopy (ATM), and polarization varying (PV)-ATM revealed that perturbing the functional state of a protein (by an inhibitor), only slightly changed the overall vibrational distribution, considered as the vibrational density of states (VDOS) (5). The same interventions, or protein mutations conferring enhanced catalytic activity, induced remarkable re-orientation of vibrational directionality despite no VDOS change (5). PV-ATM has provided dynamic fingerprinting in both proteins and nucleic acids (5, 6). Adding complexity to these molecular dynamics, many proteins, including ion channels, and redox players, act as chromophores, generating electromagnetic radiation in the form of light to execute specific signaling cascades (7, 8, 9).

Microtubules can couple both mechanical and electromagnetic waves, including light radiation, behaving as a bioelectronic circuit that provides long-range intra- and intercellular connectedness and signaling. Microtubules change their stiffness on short-time scales upon mechanical stimulation, with linear filament connections acting as transistor-like, angle-dependent momentum filters, and triangular networks behaving as stabilizing elements (10). These observations suggest that cells can tune mechano-sensing/-transduction by previously unsuspected temporal and spatial filtering flexibility. Moreover, microtubules exhibit complex, tunable electrical properties, as has been shown with the loose patch clamp technique in either the presence or absence of the microtubule stabilizer Paclitaxel (11). In this study, electrical oscillations at different holding potentials were observed, which responded accordingly in amplitude and polarity. The spectral analysis of the time records revealed a single fundamental peak at 39 Hz in the Paclitaxel-stabilized microtubules, while a more complex oscillatory response and two mean conductances were observed in the absence of the stabilizing agent. These findings point to microtubules as electrical actuators behaving as “ionic-based” transistors to generate, propagate, and amplify molecular signals.

**Figure 1:**
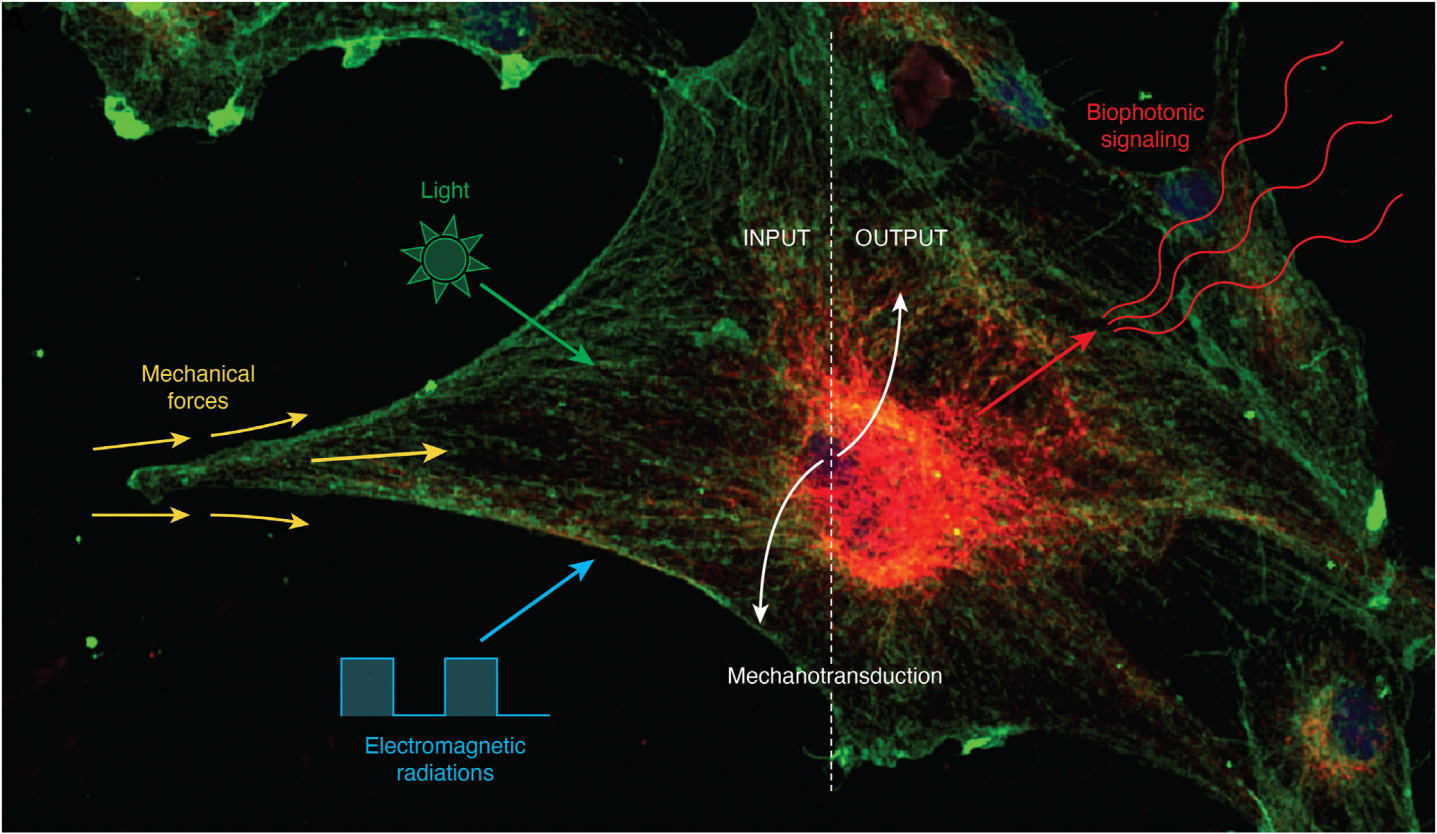
**Beyond cellular mechanotransduction.** When talking about cellular vibration, we refer to a set of stimuli (INPUT) including mechanical forces and electromagnetic radiations (light included), which can hardly be separated as they are inherent intertwined properties entailing signaling capabilities. Through mechanotransduction the cell can sense and respond to environmental stimuli, converting signals into biochemically relevant information. This process includes the deployment of a mechanical oscillation into biophotonic signaling (OUTPUT), resulting in a biophoton emission emerging from the nanomechanical vibration of proteins.

Viewing molecular vibration as an inclusive term of mechanical and electromagnetic oscillations is supported by many interrelated observations. (i) Live visualization of single isolated tubulin protein self-assembly *via* tunneling current, performed within an artificial cell-like environment, in the absence or presence of defined electromagnetic signals, provided evidence for a common frequency region in the MHz range where protein folds mechanically and its structure vibrates electromagnetically (3). The merging of electromagnetic and mechanical oscillations at a “common frequency point” has the crucial implication that we can manipulate one with another (3), suggesting future development in the refolding of misfolded proteins (as discussed below), (ii) Scanning tunneling microscopy of a single brain microtubule revealed a protein arrangement symmetry related to the conducting state written within the microtubule, showing that a memory state forms enabling the microtubule itself to behave as a memory-switching element whose hysteresis loss is nearly zero (12). These intriguing properties suggest that microtubules may play an essential role in high-speed informational processes, affecting cell biology at the level of both intra- and inter-cellular communication, (iii) Exploiting tryptophan autofluorescence lifetimes to probe energy hopping between aromatic residues in tubulin and microtubules, and investigating how the quencher concentration alters tryptophan autofluorescence lifetimes, showed that electronic energy can diffuse over 6.6 nm in microtubules (13). Such diffusion length is surprisingly high in microtubules, considering that they should be designed to play mechanical/structural roles intracellularly, and the equivalent value for chlorophyll a, which is known to be optimized for electronic energy transfer, is only in the 20 to 80 nm range (13). Diffusion lengths were influenced by tubulin polymerization state (free tubulin *versus* tubulin in the microtubule lattice), but they were not affected by the average number of protofilaments. Interestingly, exciton diffusion was decreased in the presence of anesthetics known to bind microtubules, like etomidate and isoflurane (13). These findings lead to the belief that linear and angular connections in microtubular networks may not only be involved in intracellular stiffness patterning and mechanotransduction (10), but they may be sufficient to induce long-range energy transport in tubulin polymers (13). The structural properties of microtubules have long been considered for their potential interaction with molecular machines in the development of transport-based nanodevices. The analysis of electronic energy migration in microtubules now suggests that the same protein polymeric structure of microtubules may be suitable for developing active materials in biologically sourced electronic devices where UV photoexcitation is desired. Besides these futuristic developments, the potential role of microtubules as energy-transfer cellular components raises further hypotheses on how the observed changes at a single microtubule level may coalesce into scalable bioelectronic networks within cells. A major issue will likely be understanding how the variability in mechano-electric and, possibly light-harvesting properties of microtubules, may contribute to the inherent functional variability of biological systems in an individualized manner. Biological systems entail more complexity than most physical systems, posing noticeable challenges in discriminating between coalescing classical and quantum phenomena. Within this context, the nanowire conducting properties of microtubules, and their ability to store, process, and transfer signals merging the electromechanical wave, electronic energy transfer, photons, and ionic clouds (3, 11, 12, 13) may imply that without synaptic transmission microtubules may transmit solitary waves at constant velocity without attenuation (14). According to this hypothesis, mechanotransduction in microtubules may encompass their unexpected potential to act as effective light harvesters, capable of generating and propagating multifaceted signals shaping cellular connectedness (Fig. 1). To this end, physical, and biochemical dynamics may be viewed as inputs for the bioelectronic properties of microtubules, changing the assembly/disassembly of αβ tubulin heterodimers. Accordingly, the microtubular network may be conceived as a platform for energy and information transfer, with the microtubules acting as mediators of biological quantum phenomena. These hypotheses are currently compounding the scenario for unprecedented challenges in developmental biology and medicine. These challenges include the design of adequate experimental strategies and the development of novel technologies to reconcile classical biochemistry and molecular biology with a quantum biology approach. Hopefully, understanding the quantum world of microtubules may result in the chances of improving somatic and stem cell plasticity and function, leading to unprecedented therapies for chronic diseases. In this context, an additional avenue of inquiry emerges: exploring whether signaling molecules respond to mechanical patterns, especially those of the same type that they produce. For instance, a promising issue may become the investigation of vibrational directionality at mechanical, electrical, and possibly electromagnetic levels in misfolded proteins.

Recent studies show that magnetoelectric nanomaterials can promote the dissociation of highly stable beta-amyloid (Aβ) aggregates under a low-frequency magnetic field, showing high efficacy in the clearance of microsized Aβ plaques in *ex vivo* brain tissues of Alzheimer’s disease mouse model (15). The use of physical energies to remodel vibrational directionality in misfolded proteins and restore their functionality is suggested by the observation that photobiomodulation (PBM) both reduced Aβ-stimulated microglial toxicity (16) and promoted Aβ disaggregation (16, 17). These observations are particularly attractive since, owing to their diffusive features, both electromagnetic (light included), and mechanical waves, could be exploited in tissue protein refolding *in situ*, avoiding the hurdles in the development of refolding agents and their delivery routes.

In summary, it has been shown that cellular functions and signaling are affected by long-range vibrations in proteins as well as microtubules. In a study utilizing the ATM technique, it was shown that altering protein states only slightly influenced vibrational density, while mutations could greatly change the directionality of these vibrational frequencies. The stiffness and electrical properties of microtubules depend on their abilities to serve as bioelectronic circuits that couple mechanical and electromagnetic waves. These characteristics indicate that microtubules might be most important for energy and signal transfer within cells. More interesting still is the fact that low-frequency magnetic fields have been shown to destroy β-amyloid aggregates, which may help in finding a cure for Alzheimer’s disease. Electromagnetic waves are also used in PBM to support *in situ* protein refolding and therapeutic applications.

## Photon emission and mechanical oscillations: A novel perspective on molecular signaling in normal and malignant cellular development

In the early 1990s, Albrecht-Buehler discovered that when in the absence of visible light cells were grown sparsely onto one face of a thin glass film and as a confluent layer on the opposite, they underwent a specific orientation, since the sparse cells traversed with their long axes the direction of the whorls of the confluent cells on the opposite glass face (18). Most of the orienting pattern was already visible 7 h after plating, a time at which growth of the sparse cells could be neglected, ruling out the possibility that orientation may result from the guidance of newly dividing cells. The effect was inhibited by a thin metal coating of the glass film. The author concluded that the cells were able to detect the orientation of others by signals that penetrated glass but not thin metallic films and, therefore, appeared to be carried by electromagnetic radiation. On the contrary, the cellular orienting capabilities were fully retained when the glass was coated with a thin silicone film, that allowed the transmission of radiation in the red to infrared range, indicating that cellular recognition was afforded through an electromagnetic signal carried in that range. The same author discovered that near-infrared (NIR) cellular light scattering promoted a long-range attraction essential in cell migration and aggregation (19). These studies investigated the aggregation of 3T3_x_ cells on solid substrates, where each aggregate consisted of cells that were single, and randomly placed at the time of their seeding. The experiments showed that the cells, while proliferating, were able to detect each other within a certain range and migrate to form aggregates. To prove whether light emission and scattering may be involved in the complex phenomena, hyper-scattering cells at 830 nm were prepared *in vitro*, loaded with particles of different materials, including latex, dark and white diamonds, to afford and measure tantalized, selectively controlled light scattering as a function of the type and size of the material “ingested” by the cells. The results showed that the cellular detection range became much larger than one cell diameter and was directly related to the intensities of NIR light scattering exhibited by the hyperscattering cells. The intensity of scattering (*I*_*sc*_) from hyperscattering cells exceeded the light scattering of unloaded control cells by as much as 175-fold, with an *I*_*sc*_ of particle-loaded cells passing 1000 mV, as measured by photomultiplier readings from cells that had been irradiated by the beam of an 830-nm gallium aluminum–arsenide laser (peak intensity of 40 mW). The hyperscattering cells drew closer to each other long before they were able to make physical contact, during the early few hours after plating, forming noticeable aggregates after 14 h. The results suggested that cells produced and likely modulated a NIR light scattering that mediated a long-range cellular attraction, which did not require a physical contact but was highly effective to enable cells to detect each other’s presence and modulate their collective behavior *in vitro.*

These findings pose the more general issue of understanding how the mechanics and the photonics are mutually generating each other and interacting to shape complex architectural orders in biology.

Virtually all cells have spontaneous ultraweak photon emission (UPE), unfolding in either physiological or pathological conditions (20, 21, 22, 23).

In mouse neural stem cells, and progenitor cells from the subventricular zone, UPE was assessed by a highly sensitive photomultiplier (PMT) system at the end of primary culture, at serial culture passages, before and after stem cell differentiation. The gate time for collecting the photon signal was set at 1 s. The maximum detection of PMT was 420 nm, with about 30% quantum efficiency in the range of 300 to 700 nm. The rise time for PMT was about 3 ns. By using an upper threshold, the number of counts in a dark room was detected at 1150 V, which was a relative dark count, with a subtraction of dark current. While reducing the noise significantly, this approach also caused a lower photon count per sample, which was compensated for by setting a 5-min time interval at each UPE count. Under these experimental conditions, UPE was found to significantly increase throughout subculturing passages in a neurosphere assay, at the time the neuronal differentiation took place (24). Moreover, the addition of silver nanoparticles, performed in the effort to enhance UPE, revealed that the effective increase of photon emission was paralleled with a significant enhancement in neuronal differentiation, as compared to the nanoparticle-free control cells. Intriguingly UPE after the differentiation step was found to be significantly lower than that detected before the onset of the neurogenic differentiation in both nanoparticle-free and -loaded cells (24). Owing to the technical difficulties in assessing UPE, a detailed analysis of the timely pattern of UPE scale and amplitude at each investigated step could not be provided. Nevertheless, although these findings cannot conclusively argue in favor of a causal relationship between UPE and major cell fate decisions (*i.e.*, stem cell differentiation), it appears that the observed UPE patterning may not merely reflect effector responses throughout the differentiating process. Additional studies are required to address the molecular signaling mechanisms linking UPE to the observed cellular developmental pathways.

The functional relevance of biophoton emission appears to be corroborated by studies performed with ultraweak lasers developed as “simulated biophotons” with different spectra and intensities to perform intracellular stimulation of single nerve cells of the hippocampal areas in mouse brain slices (25). The approach was designed to integrate intracellular membrane potential recording, single nerve cell intracellular ultraweak laser (simulated biophoton) stimulation, and biophoton imaging technology. Three ultraweak lasers (at red: 650 nm, green: 532 nm, and blue: 405 nm) were set at high, medium, and low intensities and regarded as simulated biophotons. Single cells were stimulated 5 min after the collection of the membrane potential signal, either with simulated green biophotons at high intensity, or with simulated red, green, and blue biophotons at the three different intensities. With this strategy, the authors could document an extremely fast (within seconds) and cumulative rise in the average intensity level of biophoton emissions, with the relative gray values (RGVs) being approximately 900, 1800, and 3600 in response to the low-, medium-, and high-intensity level of simulated biophotons, respectively. Under these conditions, the biophoton emission recorded in response to the high-level intensity was approximately 12 times the average intensity level of biophoton emission induced by 50 mM glutamate (25).

Of note, stimulation with “simulated biophotons”, generated with ultraweak lasers, elicited trans-synaptic biophotonic activities and transmission in both ipsi- and contro-lateral hippocampal projection circuits (25). Biophotonic activities and transmission, as detected by biophoton imaging, were dependent upon the spectra and intensities of the simulated biophotons but were not related to the levels of membrane potential before simulated biophoton stimulation. These findings, and the fast response in terms of biophoton emission, suggest the intervention of specific, code-dependent modulation and storage memories of neuronal signals in the form of light patterning. Unveiling the molecular players responsible for such biophotonic activities may represent a challenging subject for future studies.

Neurotransmitter-mediated chemistry can also involve biophotonic signaling. Combining an ultraweak biophoton imaging system with a biophoton spectral analysis device, allowed a spatio-temporal characterization of glutamate-induced biophotonic activities and transmission in mouse brain slices (26). Glutamate application (50 mM) elicited a fast and significant increase in biophotonic activities, starting after 30 min, peaking approximatively at 90 min, and persisting for a relatively long time (>200 min). Biophotonic activities in the corpus callosum and thalamus in sagittal brain slices mostly originated from axons or axonal terminals of cortical projection neurons. Such biophotonic activity was tightly and specifically regulated since it was blocked by oxygen and glucose deprivation, together with the application of a cytochrome c oxidase inhibitor (sodium azide), but only partly by an action potential inhibitor (TTX), an anesthetic (procaine), or the removal of intracellular and extracellular Ca^2+^. Noteworthy, the hyperphosphorylation of microtubule-associated protein tau led to a significant decrease in biophotonic activities in these two areas (26), highlighting the potential role of microtubules as light harvesters and biophotonic transmitters.

**Figure 2:**
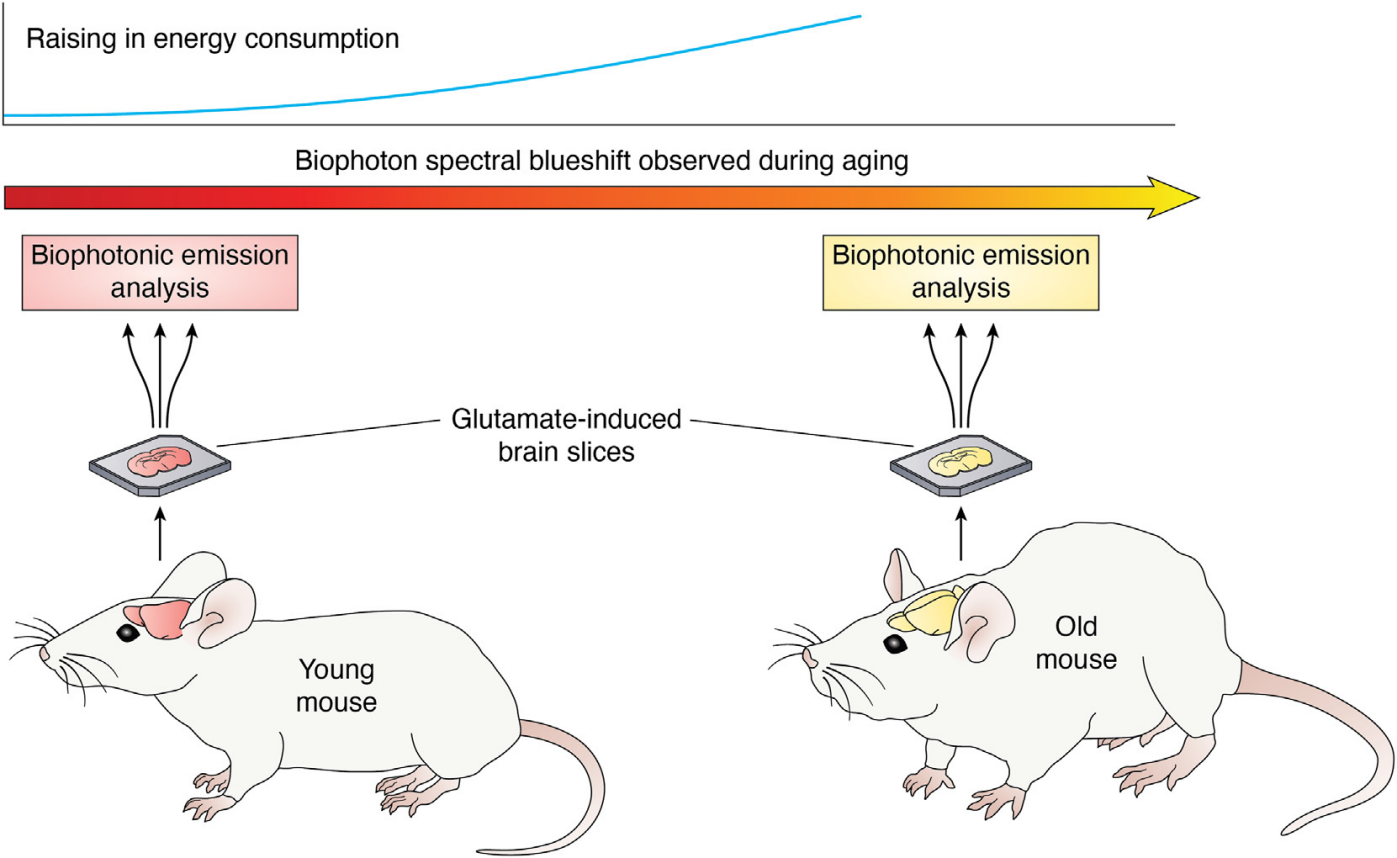
**Aging brain is associated with a spectral blueshift in biophotonic activity.** The spectral ranges of biophotons exhibit a blueshift trend during aging in mice when exposed to 50 mM glutamate (28).

The analysis of glutamate-induced biophotonic activities and transmission in the brain led to the discovery of a spectral redshift from animals (in order of bullfrog, mouse, chicken, pig, and monkey) to humans, even up to a NIR wavelength (∼865 nm) in the human brain (27). The individual differences in spectral values within the same species are very small, with nearly identical values in five bullfrogs, chickens, and mice. These observations may be a relevant biophysical cue for understanding high intelligence in humans since a biophoton spectral redshift could be viewed as a more economical and effective strategy for biophotonic signal communications coping with the increased information processing demand of the human brain (27). Accordingly, the lower to higher biophotonic redshift paralleling the increase in intelligence should be reversed in the course of cognitive decline, as it often occurs in aging processes. Confirming this expectation, the comparative analysis of glutamate-induced biophotonic emissions in mouse brain slices at different ages revealed a spectral blueshift from young to old mice (28) (Fig. 2). This finding suggests that the brain may adapt to use relatively high-energy biophotons for its computational processes during the aging process.

Specific biophotonic patterns have been detected by Chai *et al.* in mouse brain slices in response to different neurotransmitters, including acetylcholine (ACh), dopamine (DA), norepinephrine (NE), γ-aminobutyric acid (GABA), 5-Hydroxytryptamine (5-HT) and glutamate (29). The authors further refined their previous analysis of glutamate-induced biophotonic activity (25) and found that not only did it exhibit a fast (within minutes) rise but it also showed a relatively stable period of days and even presented seasonal fluctuations in a one-year-long assessment (29). These data suggested that glutamate-induced biophotonic activity and transmission may represent the underlying biophotonic information, involved in maintaining a subconsciousness state in mice, which appears to show seasonal variation, whereas altered states of consciousness may encompass the coordination and regulation of glutamate action by other neurotransmitters. To this end, the authors investigated how glutamate-induced biophotonic activity and transmission are regulated by other neurotransmitters, and they found that DA and 5-HT induced glutamate-like biophotonic activity, but with a shorter initiation period and less sustained maximum effect (29). Moreover, while a single application of ACh, NE, or GABA did not induce glutamate-like biophotonic activity, their individual application to brain slices during the maintenance period of glutamate-induced biophotonic activity elicited distinct up- or down-regulation of the glutamate response. These modulatory responses occurred with a few-minute latency and displayed a long-lasting persistence throughout several hours of observation. The observed individual or combinatorial biophotonic profiles could be finely tuned, in a dose-dependent manner in the presence of different concentrations of propofol, an agent known for inducing eyelash reflex loss at low doses, while acting as an anesthetic inducing loss of consciousness at high doses. The low-, and high-dose of propofol caused a transient or persistent inhibition in glutamate-induced biophotonic activity, respectively. In addition, while the low dose of propofol did not affect the synergistic effect of ACh, DA, and NE on the glutamate action, the same agent completely abolished these synergistic effects when employed at an anesthetic dosage (29). Notably, also the modulatory action of propofol took place within a few minutes of its administration.

These observations led to the hypothesis that biophotonic patterning may be implicated in the origin and altered states of consciousness. It has also been proposed that biophotonic signals elicited by neurotransmitters may emerge as a result of the propagation of quantum energy levels of the neurotransmitters themselves (30).

Cellular biophotonic signaling has been shown to change in response to cell perturbation by stressors and in non-cancer *versus* cancer cells. Change of culture medium *per se* induced a transient and cell-type specific UPE increase (31). Change of the culture medium in combination with the pro-inflammatory cytokine tumor necrosis factor-alpha (TNF-α) evoked a time-, and dose-dependent enhancement in UPE in both non-cancer and cancer cell lines, with UPE increasing significantly more in cancer than in non-cancer cells (31). Garcia-Montero *et al.* (32) demonstrated that a medium change elicited significant perturbations to different cell types in culture, transiently increasing the expression of p38 (peak at 60 min), JNK (peak at 15–30 min), ERK1/2 (peak at 15 min), C/EBPβ (peak at 2–3 h), and p8 (peak at 4–6 h). However, the observed increase in UPE owing to medium change alone or in combination with TNF-α occurred as an immediate response, in less than a minute time frame (at *t* = 0 min), decaying quite fast already at the second measurement (at *t* = 30 min) (31). These observations indicate that the observed UPE increase was not likely an epiphenomenon of stress-induced gene expression. The observed changes in UPE exhibited an additional trait, since they occurred as an oscillatory pattern, with the TNF-α-induced oscillations emerging as a significantly different damped signature based on the type of the cancer cell line being investigated (31). While raising the challenge of understanding the precise role of biophotonic signaling in the cellular circuitries that emerge in malignant transformation, these studies provocatively indicate that a fine oscillatory biophotonic tuning exists in response to inflammatory stress and the cancer cell microenvironment. The oscillatory nature of biophotonic signaling also poses the issue of identifying the subcellular sources for UPE, elucidating whether a major emitting center, or rather an array of multiple subcellular molecules may act as cooperating centers accountable for the detected UPE. In this regard, the discovery that biophotons may transmit along neural fibers and in neural circuits (26), and the theoretical implication of microtubule dynamics discussed herein, may lead to hypothesizing a spectral coding and quantum computation *via* microtubules based on biophotonic activity (33). Although on a speculative basis so far, the weak biophotonic intensity may not affect microtubules as quantum information carriers since, according to the current assumptions on quantum teleportation (transmission), the change of a quantum state would be permissive for information transfer if such a state is in quantum entanglement (34). A contribution by the redox environment in cell and tissue UPE patterning has also been envisioned. In particular, it has been hypothesized that changes in the culture medium or cell exposure to stressors may induce fast and transient perturbations in mitochondrial function and ROS production in association with changes in mitochondrial membrane potential (31). Dissecting the possible interplay between microtubular dynamics and cell redox state and metabolism is an intriguing field of investigation, particularly in consideration that cytoskeletal assembly and dynamics represent a major energetic drain themselves, accounting for about 50% of total ATP consumption in different cell types (1).

On the whole, the findings discussed so far indicate that, when considering the biophysical and functional sequelae of mechanical vibration, the term mechanotransduction should include the deployment of a mechanical oscillation into biophotonic signaling. As a result, mechanotransduction can be conceived as a process ensuing into a form of endogenous PBM of the molecular dynamics in living systems. The reverse path has also been shown, where exogenously applied PBM deeply impacts endogenous cellular mechanics and cytoskeletal mechanotransduction. Such interchangeable relationship was again the result of a pioneering discovery by Albrecht-Buehler, showing the first evidence that mammalian cell exposure to pulsating NIR light of 1 s pulse length reduced the stability of the radial microtubules around the centrosome, as shown by the light-induced altered resistance to nocodazole (35). These results suggested that the centrosome responded to the pulsed light by sending signals along its radial array of microtubules whose stability was then altered. These seminal discoveries have been largely confirmed in subsequent studies showing the capability of pulsed PBM to afford complex cytoskeleton remodeling and changes in cell elastic modulus, leading to increased stem cell proliferation and improved wound healing (36), as well as to changes in cell migration and morphology (37, 38). PBM-mediated cytoskeletal assembly and disassembly have also been implicated in neuroprotective responses (39). Moving further on this path will likely represent the avenue for an unprecedented understanding of some fundamental characteristics of our biology, hopefully disclosing new paradigms of cures for acute and chronic diseases.

## Biophoton signaling and photobiomodulation: Oscillatory codes for targeted molecular responses and tissue regeneration

The above-discussed findings raise the possibility of using exogenous PBM to activate endogenous biophotonic and mechanotransduction signatures through specific modulation frequencies and wavelengths.

The application of a pulsed wave (PW)-PBM at 810 nm to human dental pulp stem cells affected mitochondrial electron transport, ROS production, cell proliferation, and alkaline phosphatase (ALP), enhancing osteogenic differentiation (40). Pulsing PBM from 1 Hz up to 3 KHz resulted in significantly higher ROS production, and osteogenic differentiation with an optimal response at 300 Hz, while continuous wave (CW)-PBM did not affect the investigated parameters (40). In this study, the authors used delayed luminescence (DL), the luminescence emitted from living organisms exposed to external light, to assess the relationship between PBM effectiveness and mitochondrial activity. The results showed DL changes occurring within the first 6 s in response to PBM, based upon the pulse frequency, with 300 Hz PBM dominantly prolonging the DL pattern and enhancing ALP activity, as compared to other frequencies tested. The initial fast response, and the differential biological response, including the terminal differentiation, according to the pulsing frequency suggest a causal role of PW-PBM and that pulsing of PBM elicits specific biological responses that wouldn’t otherwise appear through CW-PBM. These considerations have profound implications in regenerative medicine, as directing the multilineage stem cell repertoire towards targeted fates becomes a remarkable therapeutic issue.

The relevance of PW-PBM is highlighted by the results yielded in a randomized, sham-controlled, double-blinded trial conducted in healthy subjects (41). A single session of NIR PW-PBM at 40 Hz, an average frequency corresponding to behaviorally-driven gamma brain oscillations, significantly increased the power of the higher oscillatory alpha, beta, and gamma frequencies, while reducing the power of the slower delta and theta frequencies in resting subjects. Network property analysis revealed that transcranial PW-PBM acted on the integration and segregation of brain networks (41). These observations indicate that PW-PBM can be used to non-invasively modulate neuronal oscillations in humans, paving the way to further clinical investigations.

**Figure 3:**
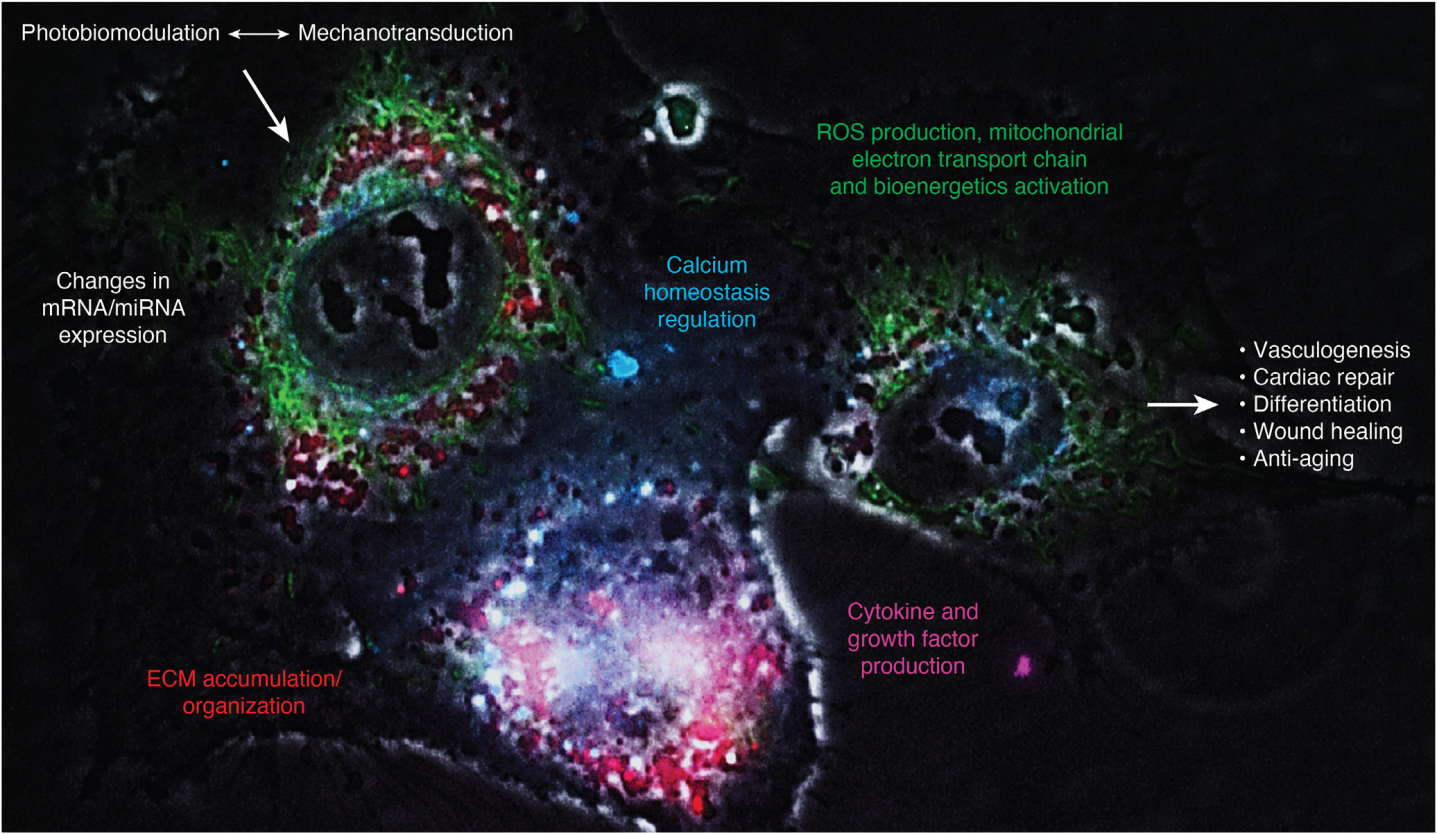
**PBM and tissue regeneration.** While mechanotransduction is a process ensuing into a form of endogenous PBM, on the other hand, exogenous PBM deeply impacts cellular mechanics and cytoskeletal mechanotransduction. In this way PBM could target the cellular mechanical and electromagnetic cues, influencing mRNA/miRNA expression, calcium homeostasis, extracellular matrix (ECM) organization, bioenergetics activation, cytokine and growth factor production, leading finally to regenerative responses (*i.e.*, vasculogenesis, cardiac repair, and wound healing).

In the cardiovascular system, vascular relaxation (photorelaxation) was induced by blue-wavelength-specific activation of melanopsin (Opn4), both *in vitro* and *in vivo* (42). In isolated mouse aortic rings, photorelaxation was wavelength-dependent, as shown by the existence of a specific light-mediated photorelaxation signature between 380 and 495 nm, and the lack of response with both the red (620–750 nm) and green light (495–570 nm). Even with blue light, photorelaxation did not occur in aortic rings from Opn4^−/−^ mice, indicating a precise chromophore target for this PW-PBM. The optimal photorelaxation with blue light was also intensity-dependent, and started within a few-second latency from PW-PBM application. Notably, photorelaxation also occurred with a few-second latency *in vivo* over a 10-min exposure to blue light of the mouse tail. A consistent increase in blood flow could be measured only in Opn4^+/+^ but not in Opn4^−/−^ mice. Overall, these data strongly argue in favor of a causal role of PW-PBM in the modulation of vascular reactivity, and prompt harnessing of the Opn4 pathway for wavelength-specific PBM-based therapies in diseases involving altered vasoreactivity and tissue perfusion. The same blue light pattern that induced vasodilation also changed cellular bioelectricity, inducing vascular hyperpolarization, as shown by intracellular membrane potential measurements (42). Photorelaxation did not involve endothelial-, nitric oxide-, carbon monoxide-, or cytochrome p450-derived vasoactive prostanoid signaling. The light-induced signaling was selectively dependent on both soluble guanylyl cyclase, and phosphodiesterase 6, which are part of a vasoreactivity network, acting as early mechano-sensors/-transducers (43). This suggests that the same molecules recruited by PBM may have turned their nanomechanical vibration into a biophotonic activity, prompting further investigation on the hypothesis discussed here that exogenous PBM may ultimately act as a form of endogenous PBM through the intervention of mechano-transducing systems. If confirmed in the future, this interplay may offer the intriguing possibility to use physical energies to target the cellular mechanical and electromagnetic cues and manipulate one with another to orchestrate defined developmental steps and regenerative responses (Fig. 3).

Dual-NIR PW-PBM, using pulsed 810 nm at 10 Hz, followed by super pulsed 940 nm at 100 Hz (10 min each) synergistically improved burn wound healing in rats by enhancing wound area contraction, cell proliferation (increased PCNA, cytokeratin-14, and TGF-β2 expression), neoangiogenesis (increased HIF-1α, CD31 expression), ECM accumulation/organization (increased type-3 collagen, fibronectin expression), dermal hydration (increased AQP3 expression), calcium homeostasis (increased TRPV3, calmodulin expression) (44). TRPV3 is a light-/thermo-sensitive Ca^2+^ permeable channel, highly expressed in skin keratinocytes, capable of activating downstream pathways promoting cell proliferation and wound healing. Compounding the mechanistic action of PW-PBM, analysis of mitochondrial activity in tissue homogenates from wounded rats revealed a progressive significant increase in cytochrome c oxidase activity (CCO) and ATP levels following PW-PBM at 810 nm and 940 nm, peaking when the combinatorial dual-NIR PBM pattern was applied. Similarly, AMP-activated protein Kinase-α (AMPK-α) was significantly more expressed in the skin during burn wound healing induced by dual-NIR PW-PBM than following exposure to 810 nm or 940 nm PW-PBM. CCO acts as the terminal component in the electron transport chain, to reduce oxygen atoms, generating ATP and water. AMPK is a crucial player energy sensor protecting cells from stress-induced ATP depletion. The fine-tuning and graduation in the activation of these bioenergetic conductors, with their synergistic upregulation owing to the combined dual-NIR PW-PBM treatment, suggests that PW-PBM may have acted to precisely activate mitochondrial dynamics to cope with the high-energy demand for cell division, migration, and differentiation during wound repair (44).

Concerning the cardiovascular system, PBM has been found to elicit post-infarct cardiac regeneration in pigs, a large-animal model of myocardial infarction (MI), closely recapitulating human MI patterns (45). NIR Ga-Al-As diode laser at 808 nm, equipped with a rigid fiberoptic, was applied for 100 s, at 30 min, 24 h, 2 days, and 7 days post MI, to both tibias and the contralateral side of the iliac bone. As early as 4 h post-MI, a significant increase in the plasmatic levels of total antioxidants was observed, persisting up to 96 h post-MI. A marked decrease in plasmatic troponin I, and creatine phosphokinase (CPK) was already evident 24 h post MI. These effects were followed by a long-lasting and remarkable decrease in the infarct size, documented at 3 months post MI. At this time point, histological analyses revealed a substantial decrease in myocardial scarring, with increased capillary density and imaging suggestive for novel cardiomyocytes (CMs) (45). The structural and functional recovery induced by the PBM treatment was corroborated by an MRI scan and ultrasound analysis.

Intriguingly, PBM elicited substantial cardiac regeneration although it was applied to the tibias and iliac bone of the animals, two very distant sites from the MI (45). Soon after 24 h post-MI a significant increase in circulating c-kit^+^ stem cells was observed in PBM-treated pigs, as compared to unexposed animals. This observation suggests that PBM likely induced stem cell mobilization from the bone marrow. Moreover, the progressive decline in circulating c-kit^+^ stem cells at 48 h post-MI was followed by a consistent increase in their number at the level of the infarct at 90 days post-MI. These findings indicate that PBM acted very rapidly in creating an immediate rescuing response, laying the foundation for subsequent stem cell recruitment at the site of injured myocardium and long-lasting tissue regeneration. The same findings also show the feasibility of myocardial rescue without the need for stem cell harvesting, isolation, expansion *in vitro*, and subsequent injection back into the heart. These clinical perspectives are further corroborated by the activation of precise transcriptional and post-transcriptional rescue mechanisms for cardiac remodeling in infarcted rats, showing that: (i) CW-PBM at 660 nm provoked a robust decrease in mRNA expression of molecules participating in post-MI inflammation and ECM composition, including IL-6, TNF receptor, TGF-β1, and collagen I and III (46). The PBM action also encompassed a decrease in post-MI expression of miR-221, miR-34c, and miR-93, which are deleterious in cardiac remodeling (46); (ii) CW-PBM added to a standard heart failure therapy provided additional improvement in cardiac remodeling in infarcted rat hearts (47); (iii) PBM, when applied after ischemia in MI in rats, reversed the changes in mRNA expression of myocardial ECM genes induced by MI, and modified cardiac miRNA expression related to fibrosis replacement in the myocardium (48).

Concerning the mechanistic bases of the protective action exerted by light radiation on the myocardial tissue, a 10-min PBM treatment at 630 nm was found to induce cell cycle re-entry *in vitro* in isolated mice neonatal CMs. This response occurred through an increased expression of miR-877-3p, followed by down-regulation of its target gene GADD45g (49). These effects were specific in nature, as PBM had no effect on other miRNAs involved in cell proliferation (miR-15b, miR-19b-3p, miR-143-3p, and miR-590-3p). These findings are particularly relevant since inducing CM proliferation is currently considered a major mechanism in cardiac regeneration, as it has been shown in our previous and recent studies (50, 51, 52). *In vivo* experiments revealed that a PBM treatment at 630 nm (2.5 mW/cm^2^) for 10 min per day for five consecutive days promoted significant cardiac regeneration in a mice model of acute MI (53), reducing the size of scarred tissue, with significant enhancement in myocardial contractility and tissue vascularization. In isolated mouse CMs, only red-PBM, but not yellow-, green- or blue-PBM enhanced cell viability. This effect was photo-power dependent and occurred as early as 10 min following the PBM application. Similarly fast responses were achieved following Human Umbilical Vein Endothelial Cell (HUVEC) exposure to red-PBM, resulting in a significant increase in neoangiogenesis after 30 min, with a progressive increase in capillary tube formation during the following 6 h of treatment, as compared to unexposed controls. The rescuing effect observed *in vivo* mainly resulted from CM proliferation, showing a positive correlation of the proliferation rate with the irradiation time. Moreover, the PBM treatment elicited a differential expression patterning of specific CM miRNAs (53). Among them, miR-136-5p was significantly down-regulated by PBM being probed as a cardiac photo-sensitive miRNA, an issue that was confirmed by induction of CM proliferation *in vitro* following CM transduction with a selective miR-136-5p inhibitor. To this end, PBM irradiation raised the expression of intracellular Ino80, a bioactive product of a target gene for miR-136-5p. After Ino80 knockdown, the proliferative action of PBM was abrogated (53). Of note, PBM treatment did not affect cardiac fibroblast proliferation *in vitro*. This observation suggests a selective restriction of PBM-induced proliferation in specific cell populations (53).

In short, it appears evident how recent studies highlight the potential of PW-PBM to activate endogenous biophotonic and mechanotransduction responses through specific frequencies and wavelengths, demonstrating that a given code corresponds to a given response. For instance, it has been observed how PW-PBM at 810 nm enhanced osteogenic differentiation in human dental pulp stem cells, with optimal effects at 300 Hz frequency, unlike CW-PBM. Similarly, a randomized trial demonstrated that PW-PBM at 40 Hz modulated brain oscillations, improving neuronal network integration. In the cardiovascular system, blue-wavelength PW-PBM induced vasodilation *via* Opn4 activation and improved burn wound healing through dual-NIR PBM. PBM also promoted cardiac regeneration post-MI, mobilizing stem cells and enhancing cardiac repair without stem cell harvesting. These studies mark a new path in the development of non-invasive strategies of cardiovascular regenerative medicine. In line with this approach is the recent finding that PBM may reverse some essential traits of cardiovascular aging.

Septic acute lung injury (ALI), following intraperitoneal lipopolysaccharide injection, resulted in cardiac dysfunction and aging with telomere shortening and decreased TRF1 gene expression, encoding an essential component of the shelterin complex of telomere maintenance. In this model, CW-PBM (808 nm) induced an increase in telomere length, also rescuing TFR1 gene expression (54). There is an indication that telomere dysfunction can contribute to critical illness outcomes (55, 56), and that telomere shortening is associated with a higher risk of ischemic heart disease, increased risk of coronary heart disease, and heart failure (57). The observed effect of PBM in a septic ALI model indicates that surveillance and maintenance of telomere integrity are important attributes of NIR light-mediated signaling, rescuing cardiac telomeres in the course of a septic model of multiorgan dysfunction.

We have previously shown that electromagnetic energy applied in the form of asymmetrically conveyed radioelectric fields was capable of reversing human mesenchymal stem cell senescence, a major contributor to the age-dependent decline of self-repairing potential (58, 59). An intriguing confirmation of the anti-senescent action of electromagnetic energy has come from a recent study that explored the effect of NIR PBM (850 nm) treatment on the cardiovascular aging and heart failure progression developing in a transgenic mice model overexpressing type 8 adenylyl cyclase (AC8). PBM reduced the age-associated increases in left ventricular mass, the left atrial dimension, and the left ventricular end-diastolic volume, while enhancing the myocardial contractility, and improving gait symmetry, an index of neuro-muscular coordination. Total TGF-β1 levels were significantly increased in circulation (serum) in AC8 following PBM treatments (60). This observation is worth noting, as PBM has been shown to activate endogenous latent TGF-β1 to promote repair and regeneration, invoking a fine-tuning within the pathophysiological roles of endogenous TGF-β1, enhancing its ability to promote tissue healing at the expense of scarring (61). Cumulatively, in the explored model of cardiovascular aging PBM led to a striking increase in the overall survival in PBM-treated AC8 mice (100%), compared to untreated AC8 mice (43%). The effects of PBM treatments, measured following a 3-months pause, persisted (60).

Aging processes, besides their detrimental consequences on the cardiovascular system, share the more general feature of a progressive loss in shape and functions of tissues and organs, favoring the onset and progression of human diseases that include cancer, stroke, heart failure, diabetes, and dementia. These outcomes pose a major threat to medical, socioeconomic, and health policies. The results yielded so far in counteracting cell and tissue senescence with various forms of PBM are emerging as novel, safe, non-invasive, and potentially personalized approaches featured to ameliorate or delay the effects of aging, significantly impacting the substantial burden of chronic diseases on the current healthcare systems.

## Concluding remarks and future perspectives

The continuous evolution of ATM, PV-ATM, and biophoton imaging technologies will provide unprecedented knowledge on the vibrational dynamics of proteins and supramolecular assemblies, connecting their inherent directional steering patterns to biophotonic activities and likely endogenous PBM. Innovation in hyperspectral imaging microscopy (HSI), coupled with the development and miniaturization of low-light detectors with enhanced capabilities, and the effort in realizing biologically inspired tools in the fields of chemiluminescence, photoluminescence, and electroluminescence will allow experimenting at the interface of technology and biological materials. This kind of innovation holds promise for “seeing” inside biological entities, without adulterating cells and tissues, exploiting deep learning and artificial intelligence approaches to analyze the acquired images. However, while the analysis of cellular nanomechanics has progressed to a considerably advanced state, the characterization of cellular biophotonic activity and its transmission beyond the physical cell/tissue boundaries are still at an earlier analytical phase of the investigation.

Although multiple observations strongly argue in favor of an interchangeable interconnection between cellular mechanics, biophoton signaling, and mechanotransduction, the currently available data still provide a partial, limited picture of such crucial interplay.

An interesting area of inquiry will be investigating the biological effects of PW-PBM modulated at frequencies in the same range as the natural vibrational frequencies detected from stem cells undergoing given differentiating pathways, such as cardiac, vascular, neuronal, and other commitments of relevance for regenerative medicine strategies. Addressing this issue would require deciphering specific cellular vibrational patterning from differentiating stem cells, for instance by the aid of AFM and/or HSI, then transforming the deflection/torsion of the AFM cantilever, or the HSI output, into defined electric potential difference patterns through which a PBM treatment could be modulated at different wavelengths. Whether these approaches may result in precise regulation of stem cell differentiation *in vitro* and efficiently enhance their regenerative potential *in vivo* remains to be established and represents a major field of investigation in our laboratory. This kind of study may also provide useful information about the chance of manipulating PBM with vibrational patterns obtained by a nanomechanical investigation of cellular characteristics. However, there is still a lack of standardization in PBM parameters, including light energies delivered, the structure of LED arrays, and laser devices used, across various *in vivo* studies, including some clinical trials, whose analysis falls apart from the purpose of the present review. Both light wavelength and the photons’ number (fluence) may significantly affect PBM efficacy. Red light (600–700 nm) displays higher photon quantum energy (2.07–1.77 eV) than NIR (808 nm = 1.53 eV), and can more easily induce tissue electrochemical changes. On the other hand, NIR light increases mostly a molecular vibrational state, which may lead to a transient thermal effect (at least 2 °C in tissues with thickness from 3.0 to 5.0 mm) and increased metabolic activity. Therefore, comparing the effects of different wavelengths should consider their photon energy, an issue that is often missing in many studies. To this end, the differentiating response of odontoblasts at different wavelengths was significantly improved when equivalent photon fluence dosing was used, suggesting that considering wavelength-specific photon energy transfer during PBM dosing could enhance clinical safety and efficacy (62).

Bearing these criticisms in mind, can further studies ensue into a new perspective of care? We believe so. All cells in our body have light receptors, using light radiation to talk to each other. Moreover, cells can use light to promote tissue regeneration, and the current data prompt the future implementation of PW-PBM “libraries” of modulation patterns, designed to direct the multilineage potential of stem cells toward targeted regenerative outcomes. It is hoped that the innovation in molecular cell biology brought by photonics science will foster a new generation of wearable devices, embedding *ad hoc* designed photo-emitting actuators to convey PW-PBM signatures to diseased tissues/organs and perform *in situ* reprogramming of tissue-resident stem cells to boost our inherent self-healing potential. The same goals could be achieved by the creation of novel living environments with expanded well-being capabilities, including ambient lighting, capable of delivering precise PW-PBM signatures, assisted with digital displays, and artificial intelligence. These approaches are still largely lying in the field of speculation. Nevertheless, we are currently facing a period of unprecedented, fast evolution in technologies designed to promote human care and life extension. In the next few years, we will eventually experiment with whether such endeavor may disclose novel perspectives in regenerative medicine.

## Data availability

All representative data are contained within the article.

## Conflict of interest

The authors declare that they have no conflicts of interest with the contents of this article.
